# Image-guided radiofrequency versus microwave ablation for small to medium hepatocellular carcinoma: a meta-analysis

**DOI:** 10.3389/fonc.2026.1830628

**Published:** 2026-07-08

**Authors:** Yujun Fu, Lin Wang, Tao Hu, Huan Liu

**Affiliations:** 1 Department Of Radiology, The Second Xiang Ya Hospital Of Central South University, Changsha, China; 2 Department of Respiratory and Critical Care Medicine, Zhuzhou Central Hospital, Zhuzhou, China

**Keywords:** image guidance, meta-analysis, microwave ablation, radiofrequency ablation, small and medium-sized hepatocellular carcinoma, systematic review

## Abstract

**Objective:**

This meta-analysis was conducted to compare the clinical efficacy and safety of image-guided radiofrequency ablation (RFA) versus microwave ablation (MWA) for the treatment of small to medium-sized hepatocellular carcinoma (HCC).

**Methods:**

A systematic search of four English databases (PubMed, Web of Science, Cochrane Library, EMBASE) was performed up to January 8, 2026. Randomized controlled trials (RCTs) and cohort studies comparing RFA and MWA for HCC were included. The Cochrane Risk of Bias tool (RoB 2.0) and the Newcastle-Ottawa Scale (NOS) were used for quality assessment of RCTs and cohort studies, respectively. Evidence certainty was evaluated via the GRADE system. Statistical analyses were performed using Review Manager 5.4 and Stata 18, with results expressed as relative risk (RR) and 95% confidence interval (CI). The study protocol was registered in the International Prospective Register of Systematic Reviews (PROSPERO) (ID: CRD420261297809).

**Results:**

Nineteen studies (6 RCTs, 13 cohort studies) were included. No statistically significant differences were observed between RFA and MWA in the complete ablation rate (RR = 1.00, 95% CI 0.98–1.01, P = 0.84), 5-year overall survival (RR = 0.99, 95% CI 0.93–1.05, P = 0.67), 1-year disease-free survival (RR = 0.97, 95% CI 0.90–1.04, P = 0.45), or incidence of major adverse events (RR = 0.93, 95% CI 0.69–1.27, P = 0.66). An initial analysis suggested a superior 1-year overall survival for MWA (RR = 0.97, 95% CI 0.95–0.99, P = 0.008), but this difference was not sustained after excluding studies with a high risk of bias (P = 0.05). Substantial heterogeneity was present for 5-year disease-free survival (I²=81%). The overall certainty of evidence was rated low per GRADE.

**Conclusion:**

For small to medium-sized HCC, image-guided RFA and MWA exhibit comparable core efficacy and safety. Treatment selection should be individualized based on tumor characteristics, institutional resources, and operator expertise. The findings concerning 5-year disease-free survival require cautious interpretation. Future large-scale, multicenter RCTs with standardized protocols are needed for definitive validation.

**Systematic Review Registration:**

https://www.crd.york.ac.uk/PROSPERO/view/CRD420261297809, identifier CRD420261297809.

## Introduction

Primary hepatocellular carcinoma (HCC) is a malignancy with globally high incidence and mortality rates, posing a serious threat to human health. In 2020, an estimated 905,700 new cases and 830,200 deaths occurred worldwide, making HCC the sixth most commonly diagnosed cancer and the third leading cause of cancer-related death (1). Among HCC cases, small-to-medium-sized HCC represents a critical stage for achieving local radical cure due to its smaller tumor volume, limited local invasion, and lower risk of distant metastasis. The efficacy of treatment at this stage directly impacts patients’ long-term survival prognosis (2). With the rapid development of minimally invasive techniques, image-guided tumor ablation has become a core treatment option for patients with small-to-medium-sized HCC who are ineligible for, cannot tolerate, or decline surgical resection. This is attributed to its advantages of minimal invasiveness, rapid recovery, confirmed efficacy, and repeatability, leading to its widespread application in clinical practice (3). Radiofrequency ablation (RFA) and microwave ablation (MWA) are currently the two most established and widely used ablation technologies.

RFA induces coagulative necrosis of tumor cells by generating heat from high-frequency ionic vibration within tissues via applied radiofrequency currents. This technique was developed earlier, has a standardized operational protocol, and boasts extensive accumulated clinical experience (4). In contrast, MWA utilizes a microwave field to cause high-speed vibration and collision of polar molecules, such as water and ions, within the tumor tissue, thereby generating heat. MWA is characterized by faster temperature rise, larger ablation zones, and less susceptibility to heat sink effects from blood perfusion. Consequently, its clinical application has been progressively increasing in recent years (5).

Several previous meta-analyses have compared the efficacy and safety of RFA and MWA for liver cancer, but most have focused on either specific study designs (e.g., only RCTs) or mixed HCC stages without specifically isolating the small-to-medium subgroup. Dou et al. (2022) reported that in cohort studies, MWA was associated with a higher complete ablation rate and lower risk of local tumor progression, although no significant differences were observed in overall or disease-free survival (6). This discrepancy may stem from the inclusion of cohort studies with variable baseline comparability. Spiliotis et al. (2021) conducted a subgroup analysis suggesting a significantly lower local tumor progression rate for MWA than RFA in HCC patients (7). However, their analysis did not specifically address the small-to-medium HCC definition. Alhasan et al. (2024) suggested that RFA might be associated with lower rates of both minor and major complications compared to MWA (8). However, that meta-analysis included liver metastases as well, potentially confounding the safety profile for HCC alone. Yu et al. (2021), analyzing RCTs for early-stage HCC, concluded that safety and efficacy were similar, with MWA requiring shorter ablation times (9). However, early-stage HCC (often BCLC 0/A) is not strictly equivalent to the small-to-medium tumor size criterion (single ≤5 cm or ≤3 nodules each ≤3 cm) used in the present study. Given these inconsistencies and limitations, the rationale for conducting the present meta-analysis was to provide an updated, comprehensive, and methodologically rigorous synthesis specifically for the small-to-medium HCC population.

Based on the current research landscape and clinical needs, this systematic review and meta-analysis aims to compare the clinical efficacy and safety of image-guided RFA versus MWA for patients with small-to-medium-sized HCC. The primary outcomes include complete ablation rate, overall survival, disease-free survival, and major adverse events. By providing an updated and rigorous evidence synthesis, this study seeks to inform clinical decision-making and guide the selection of appropriate ablation strategies in routine practice.

## Methods

### Literature screening and data extraction

#### Eligibility criteria

Inclusion Criteria: (1) Study types included randomized controlled trials (RCTs), prospective cohort studies, or retrospective cohort studies with balanced baselines and complete data. Reviews, case reports, and basic research were excluded. (2) Study participants were patients with pathologically or clinically confirmed primary small-to-medium-sized HCC, meeting the criteria of a single tumor diameter ≤5 cm, or ≤3 tumors with each diameter ≤3 cm, and without extrahepatic metastasis or severe organ dysfunction. (3) Patients had not received prior radical surgery, radiotherapy, chemotherapy, targeted therapy, immunotherapy, or other anti-tumor treatments. (4) The intervention involved one group undergoing RFA and another undergoing MWA, both under image guidance, with consistent perioperative adjuvant treatments between groups. (5) Studies reported at least one of the following outcome measures: CR, overall survival (OS), disease-free survival (DFS), or MAE.

Exclusion Criteria: Studies were excluded if they were non-comparative, involved participants not meeting the definition of small-to-medium-sized HCC, had unclear interventions, contained incomplete or non-extractable data, were duplicate publications, conference abstracts, unpublished dissertations, or full texts were unavailable.

Study Classification and Analysis: Two investigators independently screened the literature and extracted data. Discrepancies were resolved through discussion or adjudication by a third reviewer. Included studies were categorized by design into an RCT subgroup and a cohort study subgroup for corresponding subgroup and pooled meta-analyses.

#### Information sources

To identify eligible clinical studies, this systematic review and meta-analysis conducted computerized searches of four core English databases. The last search date for all databases was January 8, 2026. Search operations were performed independently by two investigators, with results cross-checked and discrepancies resolved through consensus.

The specific databases searched were: PubMed, Web of Science, Cochrane Library, and EMBASE. Furthermore, after the initial literature screening, the reference lists of included studies and relevant systematic reviews/meta-analyses were manually reviewed to supplement potentially missed eligible studies. Searches were limited to the English language. No publication type restrictions were applied *a priori*. Screening was conducted strictly based on the pre-defined eligibility criteria to minimize publication bias and the risk of missing relevant literature.

### Search strategy

All database searches were executed independently by two investigators, with the last search uniformly conducted on January 8, 2026. Searches were limited to the English language. The search strategy combined Medical Subject Headings (MeSH) terms and keywords, including: hepatocellular carcinoma, HCC, microwave ablation, MWA, radiofrequency ablation, RFA, complete ablation rate, local tumor progression, overall survival, disease-free survival, adverse event. The detailed search strategy (including search strings and field settings for each database) is provided in **Supplementary Table 1**. Additionally, we manually screened the reference lists of included studies and relevant review articles to capture potentially eligible studies missed by the electronic database searches. This systematic review and meta-analysis was conducted in strict accordance with the Preferred Reporting Items for Systematic Reviews and Meta-Analyses (PRISMA) statement (5). The protocol for this meta-analysis was registered prospectively on the International Prospective Register of Systematic Reviews (PROSPERO) platform, and followed the PRISMA-P guidelines.

### Study selection process

Study selection was performed independently and in duplicate by two trained investigators using a pre-defined, stepwise process based on the eligibility criteria. After removing duplicates in EndNote 21, both investigators screened titles and abstracts, followed by full-text assessment. Discrepancies at any stage were resolved by discussion or by a third senior investigator. EndNote 21 was used for reference management.

### Data collection process

Data collection was performed independently by two trained investigators using a standardized data extraction form in Microsoft Excel. The collected data included study characteristics, baseline patient information, intervention details, outcome measures, and methodological quality. EndNote 21 was used for literature management. Discrepancies were resolved by discussion or by a third senior investigator. Missing data were not imputed; corresponding authors were contacted when necessary.

### Data items

This study collected data for four pre-specified core outcome measures. All available measurement results and data at corresponding follow-up time points for these four outcomes in each study were extracted completely, without selective filtering.

Complete Ablation Rate (CR): Defined as the proportion of patients with no residual tumor on post-procedural imaging, indicating complete coverage of the tumor by the ablation zone.

Overall Survival (OS): Defined as the proportion of patients surviving from the initiation of intervention to death from any cause. Data reported at various follow-up time points were extracted.

Disease-Free Survival (DFS): Defined as the proportion of patients free from tumor recurrence, metastasis, or death from the initiation of intervention. Results at corresponding follow-up time points were recorded.

Major Adverse Events (MAE): Defined as adverse events requiring invasive intervention, leading to prolonged hospitalization, disability, or death. The number of events and incidence rates were extracted.

Additionally, the following variables were collected: first author, publication year, study design, sample sizes of both groups; patient baseline characteristics including age, gender, liver function Child-Pugh grade, tumor number, tumor size, and whether patients were treatment-naive; intervention details including image guidance modality and core procedural techniques for both RFA and MWA; and the study follow-up duration.

### Bias and evidence quality assessment

#### Risk of bias in individual studies

The Cochrane Risk of Bias tool for randomized trials, version 2 (RoB 2.0), was used to assess the risk of bias in included RCTs. This tool systematically evaluates five core domains: the randomization process, deviations from intended interventions, missing outcome data, measurement of the outcome, and selection of the reported result. The risk of bias for each RCT was independently assessed by two methodology-trained investigators and judged as ‘low’, ‘some concerns’, or ‘high’ for each domain, following the standardized RoB 2.0 criteria.

The Newcastle-Ottawa Scale (NOS) was used to assess the quality of included cohort studies. The NOS comprises eight items across three domains: selection of study groups (4 points), comparability of groups (2 points), and ascertainment of the outcome/exposure (3 points), with a maximum score of 9 points. Two investigators independently assessed each cohort study. Based on predefined cut-off values, studies with NOS scores of 7–9 were classified as high quality, 5–6 as moderate quality, and ≤4 as low quality, in line with previous meta-analyses (10, 11).

After independent assessments, results were cross-checked. Discrepancies were resolved by discussion or by a third senior investigator. A standardized assessment form in Microsoft Excel recorded all judgments.

#### Reporting bias

This study employed multiple methods to assess the risk of publication bias and selective outcome reporting. For each core outcome, funnel plots were generated for qualitative visual assessment of asymmetry in the scatter plot of effect estimates, providing an initial indication of potential bias from unpublished negative results. Furthermore, Egger’s linear regression test was performed for quantitative analysis to determine if funnel plot asymmetry was statistically significant, further verifying the potential presence of publication bias.

#### Certainty of evidence

The Grading of Recommendations Assessment, Development and Evaluation (GRADE) approach was used to assess the certainty of evidence for each outcome. Two investigators independently performed GRADE assessments. Discrepancies were resolved by discussion or by a third senior investigator.

The initial certainty rating was determined by study design: high for RCTs and low for observational studies. The rating was then downgraded or upgraded based on five domains: risk of bias (based on RoB 2.0 and NOS assessments), inconsistency (significant heterogeneity in effect estimates), indirectness (differences in population, intervention, comparison, or outcome), imprecision (wide confidence intervals or small sample size), and publication bias (suggested by funnel plots or Egger’s test). Upgrading was considered for large effect sizes or evidence of a dose-response relationship. The final certainty of evidence for each outcome was categorized as high, moderate, low, or very low.

### Effect measures and synthesis methods

#### Effect measures

For all pre-specified dichotomous outcomes (CR, OS, DFS, MAE incidence), the relative risk (RR) was used as the uniform effect measure for pooled analysis and presentation. The RR, along with its 95% confidence interval (CI), was calculated to reflect the ratio of the event risk in the MWA group compared to the RFA group. Statistical significance was determined based on the 95% CI.

#### Synthesis methods

1. The core characteristics (interventions, participants, outcomes, study design) of all included studies were compiled into standardized tables. Studies were included in the meta-analysis for a specific outcome only if they compared RFA vs. MWA, reported the pre-specified outcome (CR, OS, DFS, or MAE), and had complete, extractable data.
2. Effect sizes were calculated using event counts and total sample sizes. Missing summary statistics were not imputed, estimated, or transformed.
3. For data presentation, standardized tables summarized basic information, sample sizes, and event counts for included studies. Forest plots were used to visually display individual study effect sizes, weights, and pooled results. Funnel plots were used to display the distribution of effect sizes for publication bias assessment.
4. Meta-analysis was performed for eligible outcomes using RR as the effect measure. Statistical heterogeneity was assessed using the Q-test and I² statistic. If I² < 50%, indicating low heterogeneity, a fixed-effect model was used for pooling. If I² ≥ 50%, indicating substantial heterogeneity, a random-effects model was employed to account for between-study variation and ensure robust results. Statistical analyses were performed using Review Manager (RevMan) version 5.4 and Stata version 18 software.
5. To explore potential sources of statistical heterogeneity, pre-specified subgroup analyses were planned based on study design (randomized controlled trials versus cohort studies), tumor size (≤3 cm versus >3 cm), Child-Pugh class (A versus B/C), and ablation technology type (conventional versus modified). Pooled effect sizes and heterogeneity within subgroups were compared. However, due to insufficient data reporting across included studies, only subgroup analysis by study design could be performed for the outcomes of disease-free survival.
6. Sensitivity analyses were performed to assess the robustness of the pooled results. Methods included sequentially removing individual studies and re-analyzing the data, and excluding studies with lower methodological quality. The stability of the core findings was evaluated by observing the direction, magnitude, and statistical significance of the pooled effect sizes under different analytical scenarios.

## Results

### Study selection results

**Figure 1:**
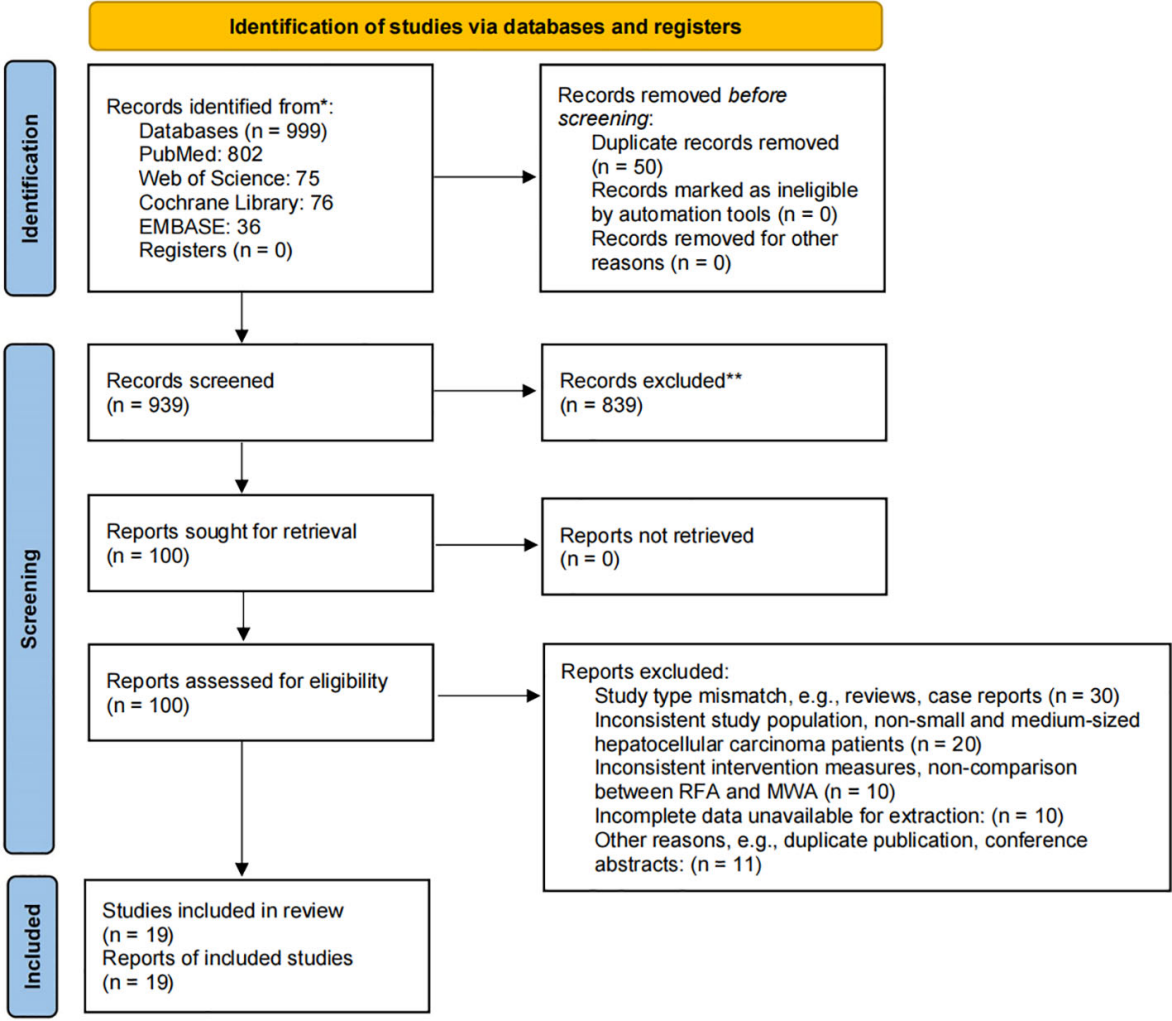
Flow diagram of literature screening.

The study selection process followed the PRISMA 2020 statement. A total of 989 records were initially identified. After removing 50 duplicates, 939 records underwent title/abstract screening. Subsequently, 839 records were excluded, and the full texts of 100 records were retrieved for eligibility assessment. Of these, 81 records were excluded for reasons such as ineligible study design or unsuitable patient population. Ultimately, 19 studies meeting the eligibility criteria were included (**Figure 1**).

A total of 19 articles were included, comprising either randomized controlled trials (RCTs) or cohort studies (**Table 1**).

**Table 1:** Characteristics of included studies.

Study (Year)	Country	Type	Imaging guidance	Modality		Number of participants		Age (years)		Female/total		Child-Pugh(A/B/C)		Tumor lesions		Tumor size (mm)		Follow-up (months)	
				RFA	MWA	RFA	MWA	RFA	MWA	RFA	MWA	RFA	MWA	RFA	MWA	RFA	MWA	RFA	MWA
Abdelaziz 2014 (12)	Egypt	RCT	US	Conv.	Conv.	45	66	56.8 ± 7.3	53.6 ± 5	14/45	18/66	24/21/0	25/41/0	52	76	2.95 ± 1.03	2.9 ± 0.97	ND	ND
An 2021 (13)	China	Cohort	CT/US	Conv.	Conv.	70	74	57.4 ± 10.1	56.4 ± 11.5	6/70	13/74	68/2/0	74/0/0	70	74	2.1 ± 0.6	2.0 ± 0.5	3.89(3.4-83.9)	37.6(3.2-79.2)
Bahloul 2026 (14)	France	Cohort	CT/US/CT-US	mbp-RFA	Single-needle	242	120	66.33(59.3-72.2)	65.52(59.1-71.7)	31/242	23/120	211/31/0	99/21/0	323	168	ND	ND	Median 27	Median 27
Chong 2020 (15)	China	RCT	CT/US	Conv.	Conv.	46	47	64.59(42.0-85.0)	63.0(50.0-80.0)	8/46	17/47	40/6/0	39/7/1	46	47	Median 2.8	Median 3.1	33.9(4.9-72.7)	38.3(2.3-78.0)
Ding 2013 (16)	China	Cohort	US	Conv.	Conv.	85	113	58.64 ± 8.52	59.06 ± 11.68	17/85	28/113	49/36/0	75/38/0	98	131	2.38 ± 0.81	2.55 ± 0.89	27.69 ± 15.28	18.32 ± 9.31
Kamal 2019 (17)	Egypt	RCT	US	Conv.	Conv.	28	28	ND	ND	6/28	7/28	22/6/0	22/6/0	34	34	3.28 ± 0.91	3.25 ± 0.92	ND	ND
Kim 2026 (18)	Korea	Cohort	US-MRI/CT	NT-RFA	Conv.	144	160	65.0(59.0-72.0)	67.0(58.0-73.0)	35/144	43/160	ND	ND	144	160	1.6(1.3-2.1)	1.6(1.3-2.0)	40.0(3.3-40.0)	40.0(3.3-40.0)
Liu 2018 (19)	China	Cohort	US	Conv.	Conv.	436	126	56(46-65)	54(45.25-60)	45/436	12/126	ND	ND	482	162	2.3(1.8-3.0)	2.25(1.7-2.9)	34.1(1-171)	36.8(1-115)
Potretzke 2016 (20)	USA	Cohort	CT/US	Conv.	Conv.	55	99	62(23-88)	61(44-82)	15/55	18/99	ND	ND	69	136	Mean 2.4	Mean 2.2	31(1-148)	24(1-57)
Qian 2012 (21)	China	Cohort	US	Conv.	Modified cooled-shaft antenna	20	22	56 ± 11	52 ± 12	1/20	2/22	20/0/0	22/0/0	20	22	2.0 ± 0.5	2.1 ± 0.4	5.1 ± 1.3	5.1 ± 1.3
Shibata 2002 (22)	Japan	RCT	US	Conv.	Conv.	36	36	63.6(44-83)	62.5(52-74)	12/36	12/36	21/15/0	19/17/0	48	46	2.3(1.0-3.7)	2.2(0.9-3.4)	Mean 18	Mean 18
Simo 2011 (23)	USA	Cohort	US	Conv.	Conv.	22	13	58.00 ± 8.64	59.63 ± 6.75	3/22	6/13	12/7/3	7/6/0	27	15	2.35(1.2-4.4)	2.31(1.4-3.9)	Mean 19	Mean 7
Sparchez 2019 (24)	Romania	Cohort	US	Conv.	Conv.	44	17	60.18 ± 9.96	62.12 ± 10.73	21/44	8/17	ND	ND	62	20	2.5(1.65-3.0)	2.55(1.5-3.3)	ND	ND
Suwa 2021 (25)	Japan	Cohort	US	Conv.	Conv.	72	72	74.40 ± 9.19	74.90 ± 8.43	23/72	25/72	61/11/0	58/14/0	86	86	1.76 ± 0.63	1.77 ± 0.68	Median 35.5	Median 11.0
Vietti Violi 2018 (26)	France, Switzerland	RCT	CT/US	Conv.	Conv.	73	71	65(59-73)	68(60-72)	11/73	12/71	53/20/0	57/14/0	104	98	1.80 ± 0.71	1.80 ± 0.65	25(18-34)	26(18-29)
Xu 2017 (27)	China	Cohort	US	Conv.	Cooled-shaft antenna	159	301	54.0 ± 11.0	54.2 ± 11.0	27/159	66/301	140/19/0	278/23/0	159	301	1.7 ± 0.3	1.7 ± 0.3	62.0(6.0-120.0)	53.0(8.0-98.0)
Yu 2017 (28)	China	RCT	US	Conv.	Conv.	200	203	ND	ND	ND	ND	ND	ND	251	265	2.6 ± 1.0	2.7 ± 1.0	35.2(2.0-81.9)	35.2(2.0-81.9)
Yu 2025 (29)	China	Cohort	CT/US	Conv.	Conv.	234	81	56(50-63)	59(52-66)	58/234	20/81	105/121/5	40/40/1	234	81	2.1(1.5-2.8)	2.5(1.9-3.4)	Median 40	Median 40
Zhang 2013 (30)	China	Cohort	US	Conv.	Cooled-shaft antenna	78	77	54.0 ± 10.5	54.0 ± 9.5	14/78	10/77	78/0/0	77/0/0	93	105	2.3 ± 0.4	2.2 ± 0.4	26.3(7.0-65.6)	24.5(6.0-64.0)

### Results of risk of bias in individual studies

#### Risk of bias in RCTs

**Figure 2:**
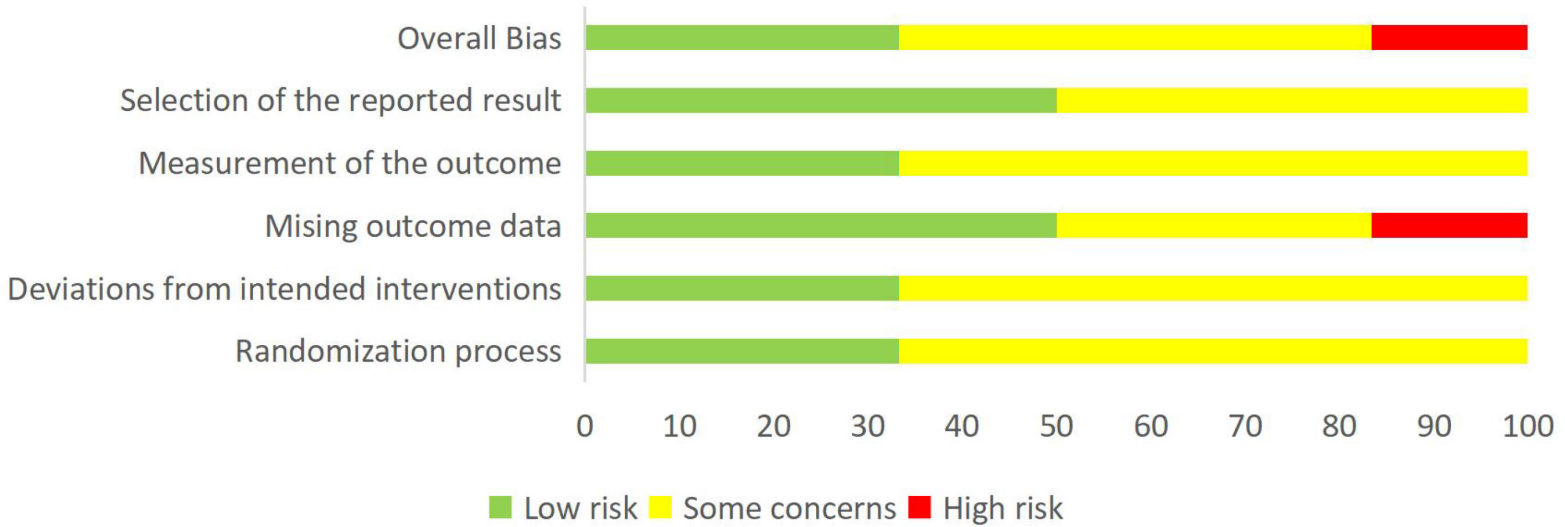
Risk of bias results for RCTs.

The overall risk of bias assessed by RoB 2.0 for the six RCTs was primarily low to moderate. However, Abdelaziz 2014 (12) was rated as high risk in the domain of ‘Missing outcome data’ due to a substantial loss to follow-up rate of 52.2% (**Figure 2**).

Regarding the ‘Randomization process’, Chong 2020 and Vietti Violi 2018 (15, 26) demonstrated low risk because of their documented proper sequence generation and allocation concealment. In contrast, the remaining four RCTs were judged as having some concerns due to insufficient detail on allocation concealment or randomization methods.

For ‘Deviations from intended interventions’, Chong 2020 and Vietti Violi 2018 (15, 26) were classified as low risk owing to their blinding procedures and low attrition. The other four studies, which lacked blinding but did not exhibit major protocol deviations, were rated as having some concerns.

In the domain of ‘Measurement of the outcome’, Chong 2020 and Vietti Violi 2018 (15, 26) were again deemed low risk due to standardized measurement protocols and blinded outcome assessment. Conversely, the remaining four studies, which lacked blinded assessment, were considered to have some concerns.

For ‘Selection of the reported result’, Chong 2020, Vietti Violi 2018, and Yu 2017 (15, 26, 28) were rated low risk, as their outcomes were pre-specified in trial registries and consistently reported. The other three studies, which did not mention pre-registration but showed no evidence of selective reporting, were judged as having some concerns. Importantly, no study was assessed as high risk in any domain due to selective reporting or critical measurement flaws.

#### Risk of bias results for cohort studies

All 13 included cohort studies achieved a high methodological quality based on the NOS, with scores ranging from 7 to 9 points (**Table 2**). These studies demonstrated well-defined representativeness of the target populations, strict adherence to predefined inclusion and exclusion criteria, and clear definitions of both exposure and outcome measures. Objective assessment methods, such as imaging examinations and pathological verification, were employed for outcome evaluation. Comparability between groups was generally satisfactory. Several studies actively balanced baseline confounders using Propensity Score Matching (PSM). The remaining studies achieved sufficient baseline comparability through natural equilibrium. The follow-up processes were complete, with durations adequate for efficacy and safety assessments. No substantial loss to follow-up bias was observed, thereby ensuring data reliability. Consequently, all studies met the core methodological quality requirements for inclusion in the meta-analysis.

**Table 2:** Risk of bias assessment scores for cohort studies.

References	a	b	c	d	e	f	g	h	Score	Overall of quality	Explanation
An 2021 (13)	1	1	1	1	2	1	1	1	9	High	Multicenter target population; IPTW adjusted for confounders; objective exposure/outcome assessment; adequate follow-up duration; no significant loss to follow-up
Bahloul 2026 (14)	1	1	1	1	2	1	1	1	9	High	Dual-center, treatment-naïve HCC population; IPTW balanced baseline; clear definition of both ablation techniques; standardized outcome adjudication; complete follow-up
Ding 2013 (16)	1	1	1	1	1	1	1	1	8	High	Single-center population meeting Milan criteria; baseline naturally balanced (no active matching/weighting); all other criteria met; adequate follow-up duration
Kim 2026 (18)	1	1	1	1	2	1	1	1	9	High	Multicenter design; comparable source for exposed/unexposed groups; clear exposure definition; PSM matched baseline; objective outcome assessment; sufficient follow-up
Liu 2018 (19)	1	1	1	1	2	1	1	1	9	High	Population meeting Milan criteria; PSM balanced baseline; clear exposure/outcome definitions; complete and adequate follow-up
Potretzke 2016 (20)	1	1	1	1	1	1	1	1	8	High	Single-center HCC population with good representativeness; no significant baseline imbalance between groups; objective exposure/outcome assessment; complete follow-up data
Qian 2012 (21)	1	1	1	1	1	1	1	1	8	High	Clear HCC target population; comparable baseline between groups; standardized exposure/outcome adjudication; complete follow-up; mean follow-up of 5.1 months sufficient for short-term efficacy assessment
Simo 2011 (23)	1	1	1	1	1	1	1	1	8	High	Clear target population; both groups from the same center; outcome verified by imaging and pathology; no significant loss to follow-up
Sparchez 2019 (24)	1	1	1	1	1	1	1	1	8	High	Population with unresectable liver metastases meeting indications; baseline naturally balanced; standardized exposure/outcome adjudication; cases with short follow-up excluded to ensure completeness
Suwa 2021 (25)	1	1	1	1	2	1	1	1	9	High	Single-center HCC population meeting Milan criteria; PSM matched baseline; clear exposure/outcome definitions; complete follow-up data; adequate follow-up duration
Xu 2017 (27)	1	1	1	1	1	1	1	1	8	High	Clear HCC target population; balanced baseline characteristics between groups; clear exposure definition; outcome objectively determined by imaging; sufficient follow-up
Yu 2025 (29)	1	1	1	1	2	1	1	1	9	High	HCC population meeting indications; PSM balanced baseline confounders; standardized exposure/outcome adjudication; complete follow-up; sufficient for long-term efficacy assessment
Zhang 2013 (30)	1	1	1	1	1	1	1	1	8	High	Well-represented HCC population; both groups from the same center; objective exposure/outcome assessment. Baseline was comparable except for tumor number; adequate follow-up duration with no significant loss to follow-up

The evaluation items are as follows: a) Representativeness of the exposed cohort for the target population, b) Selection of the non-exposed cohort, c) Ascertainment of exposure, d) Demonstration that the outcome of interest was not present at the start of the study, e) Comparability of cohorts based on key confounding factors, f) Assessment of outcome, g) Sufficient length of follow-up for outcomes to occur, h) Adequacy of follow-up.

### Results of individual studies and synthesis results

#### Complete ablation rate

**Figure 3:**
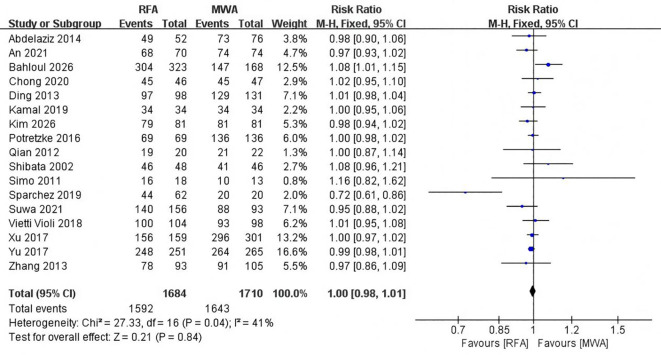
Forest plot for complete response (CR).

Seventeen studies reported complete ablation rates (**Figure 3**). The pooled RR was 1.00 (95% CI: 0.98, 1.01; P = 0.84), indicating no significant difference between RFA and MWA. Moderate heterogeneity was observed (I²=41%, Chi²=27.33, df=16, P = 0.04). Sensitivity analysis showed that exclusion of Sparchez 2019 (24) reduced heterogeneity to I²=8% without materially changing the pooled effect (RR = 1.00, 95% CI: 0.98, 1.01). Thus, the two techniques achieved comparable complete ablation rates, and the result was robust.

#### Short-term survival outcomes

**Figure 4:**
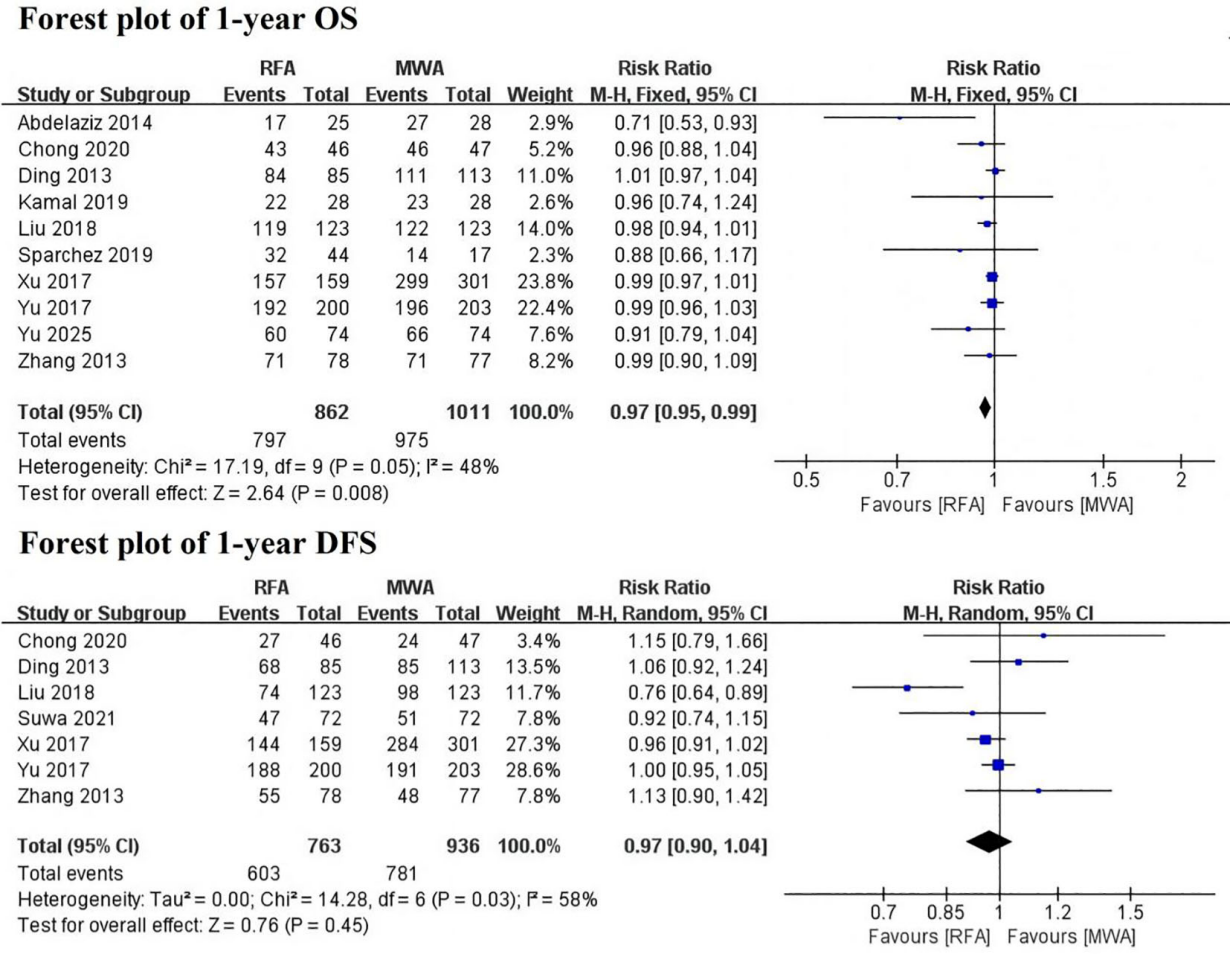
Forest plot of short-term survival outcomes.

Ten studies reported 1-year OS. The pooled RR was 0.97 (95% CI: 0.95, 0.99; P = 0.008), suggesting a statistically significant advantage for MWA. Moderate heterogeneity was observed (I²=48%, Chi²=17.19, df=9, P = 0.05). Sensitivity analysis revealed that exclusion of Abdelaziz 2014 (12)—a study with high risk of bias—reduced heterogeneity to I²=0% and changed the pooled RR to 0.98 (95% CI: 0.96, 1.00; P = 0.05). After this exclusion, the confidence interval crossed 1, indicating that the initial significant difference was not robust. Therefore, no significant difference in 1-year OS was found between the two techniques after accounting for bias (**Figure 4**).

Seven studies reported 1-year DFS. The pooled RR was 0.97 (95% CI: 0.90, 1.04; P = 0.45), indicating no significant difference between RFA and MWA. Moderate-to-high heterogeneity was present (I²=58%, Chi²=14.28, df=6, P = 0.03). Sensitivity analysis showed that exclusion of Liu 2018 (19) reduced heterogeneity to I²=3% without changing the direction of the pooled effect (RR = 0.99, 95% CI: 0.94, 1.04). Thus, the two techniques demonstrated comparable 1-year DFS, with the heterogeneity largely attributable to a single study.

#### Long-term survival outcomes

Eight studies reported 5-year OS. The pooled RR was 0.99 (95% CI: 0.93, 1.05; P = 0.67), indicating no significant difference between RFA and MWA. Moderate heterogeneity was observed (I²=47%, Chi²=13.24, df=7, P = 0.07). Sensitivity analysis showed that exclusion of Yu 2025 (29) reduced heterogeneity to I²=36% without changing the pooled effect (RR = 1.00, 95% CI: 0.95, 1.05). The two techniques provided comparable long-term overall survival.

**Figure 5:**
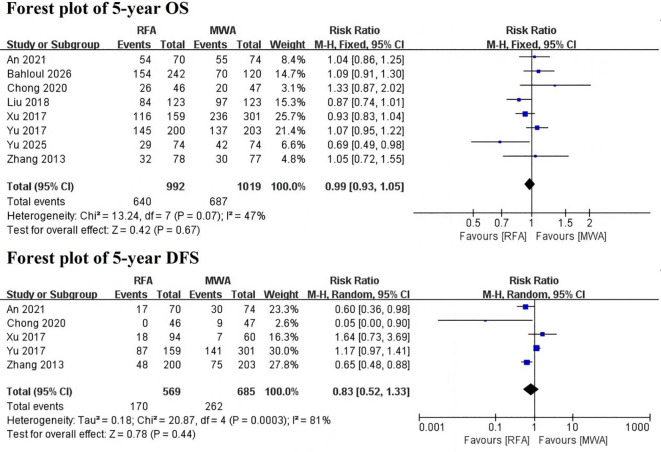
Forest plot for long-term survival outcomes.

Five studies reported 5-year DFS. The pooled RR was 0.83 (95% CI: 0.52, 1.33; P = 0.44), indicating no statistically significant difference between RFA and MWA. However, substantial heterogeneity was present (I²=81%, Chi²=20.87, df=4, P = 0.0003). Sensitivity analysis showed that heterogeneity remained high (I² range 68%–84%) regardless of which study was excluded, and no single source of heterogeneity could be identified. Given the limited number of studies and high heterogeneity, this finding should be interpreted with caution and considered exploratory. No firm clinical conclusion can be drawn for 5-year DFS (**Figure 5**).

### Major adverse events

**Figure 6:**
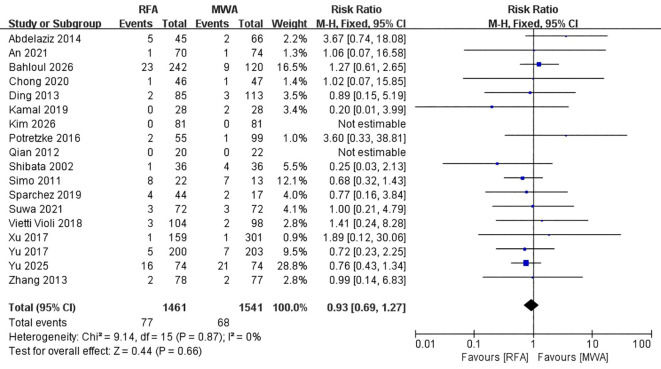
Forest Plot for MAEs.

The analysis of MAEs included 17 studies comparing RFA and MWA. Among these, two studies were excluded from the pooled effect estimate calculation because no events were recorded (**Figure 6**). Regarding individual study results, the 95% CI of the RR for every study crossed 1. This finding indicates no statistically significant difference in MAE risk between the two groups at the individual study level. Several studies exhibited wide confidence intervals. For instance, Abdelaziz 2014 (12) reported an RR of 3.67 (95% CI: 0.74, 18.08), and Potretzke 2016 (20) reported an RR of 3.60 (95% CI: 0.33, 38.81). These wide intervals primarily resulted from low event counts, which reduced estimation precision. Nevertheless, these results still did not demonstrate a clear difference between the groups.

The pooled analysis yielded a combined RR of 0.93 (95% CI: 0.69, 1.27). This result suggests no statistically significant difference in the risk of MAEs between the RFA and MWA groups (Z = 0.44, P = 0.66). Heterogeneity testing revealed no significant heterogeneity among the included studies (Chi²=9.14, df=15, P = 0.87, I²=0%). Sensitivity analysis, performed by sequentially omitting each study, consistently showed an I² of 0% without altering the direction of the pooled effect. Consequently, these findings confirm that the two ablation techniques carry a comparable risk for MAEs. The absolute event rate was 5.27% in the RFA group and 4.41% in the MWA group, with an absolute risk difference of 0.86%.

### Results of reporting bias

**Figure 7:**
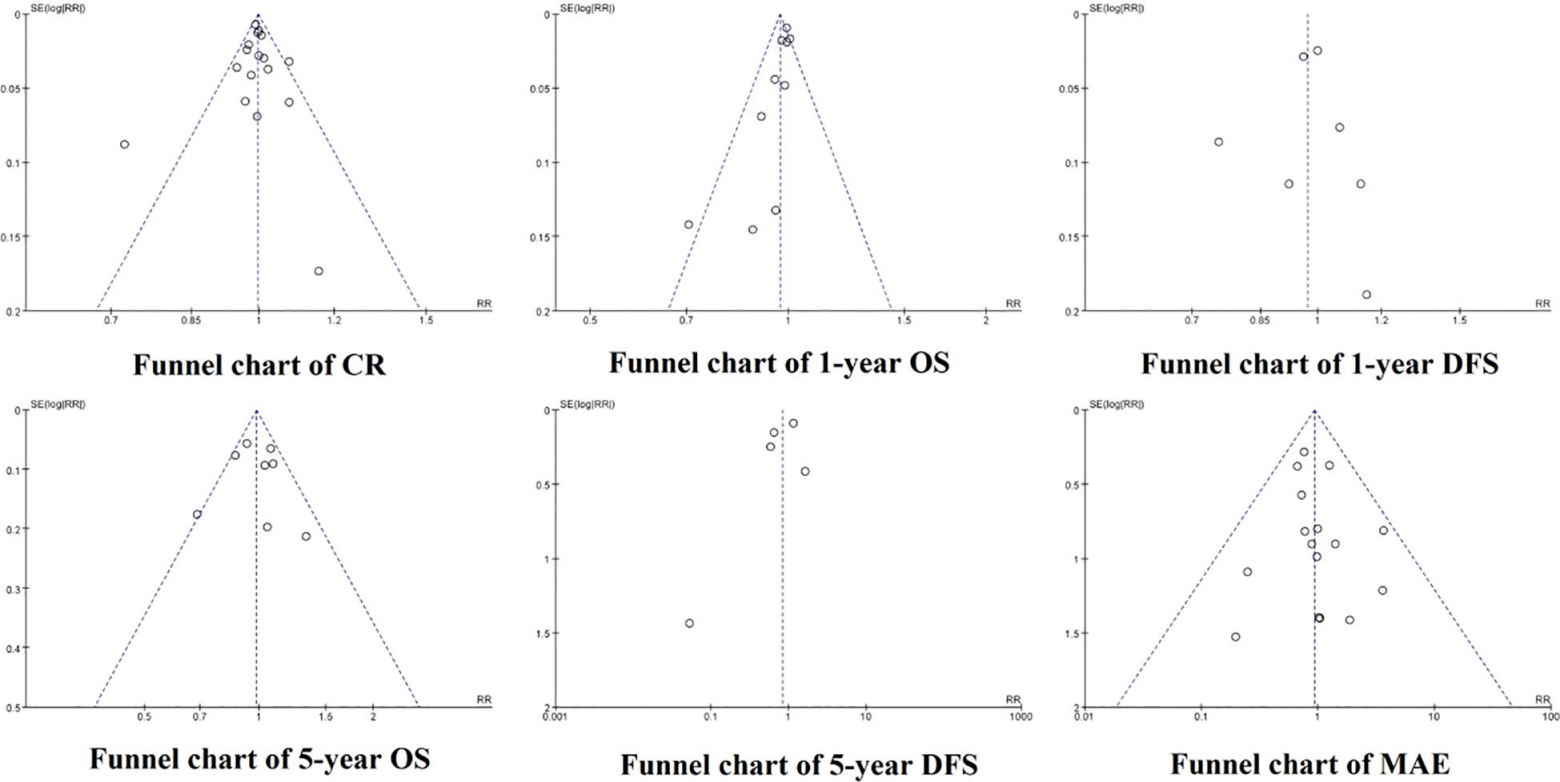
Funnel Plot of the Meta-analysis.

Publication bias was assessed using funnel plots and Egger’s tests. For complete response (CR), 1-year DFS, 5-year OS, and MAEs, funnel plots were symmetric and Egger’s tests were non-significant (all P>0.05), indicating no substantial publication bias. For 1-year OS, the initial funnel plot showed asymmetry (Egger’s test P = 0.0193), but this small-study effect was no longer significant after excluding the high-risk study by Abdelaziz 2014 (P = 0.1471). For 5-year DFS, the funnel plot was asymmetric and Egger’s test was significant (P = 0.0381), suggesting potential publication bias; however, given the limited number of studies (n=5), this result should be interpreted with caution. Overall, publication bias was not a major concern for most outcomes. (**Figure 7**).

### Subgroup analysis

**Figure 8:**
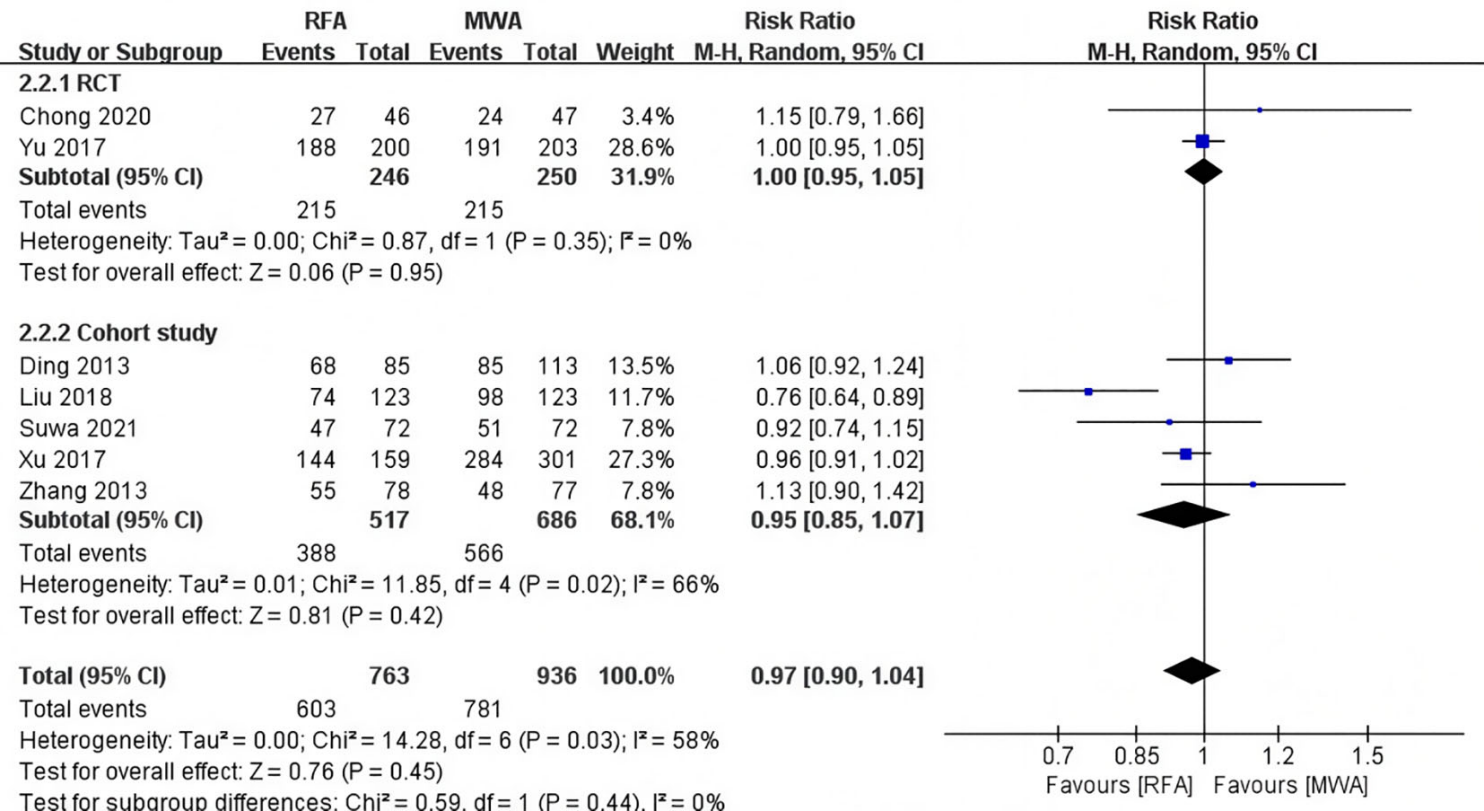
Subgroup analysis of 1-year DFS.

Due to the limited number of studies reporting DFS and insufficient data for other pre-specified subgroups, subgroup analysis was performed only based on study design (RCTs versus cohort studies) For 1-year DFS (**Figure 8**), the RCT subgroup comprised two studies. The pooled results demonstrated no significant difference between the treatment groups, with negligible heterogeneity within this subgroup (I²=0%, P = 0.35). The test for overall effect was P = 0.95. The cohort study subgroup included five studies. Similarly, the pooled analysis for this subgroup revealed no statistically significant difference in the 1-year outcome. However, moderate heterogeneity was present within this subgroup (I²=66%, P = 0.02). The overall pooled analysis combining both subgroups continued to show no significant difference, with moderate overall heterogeneity. The test for subgroup differences indicated no statistically significant variation in effect estimates between the different study designs (P = 0.44, I²=0%).

**Figure 9:**
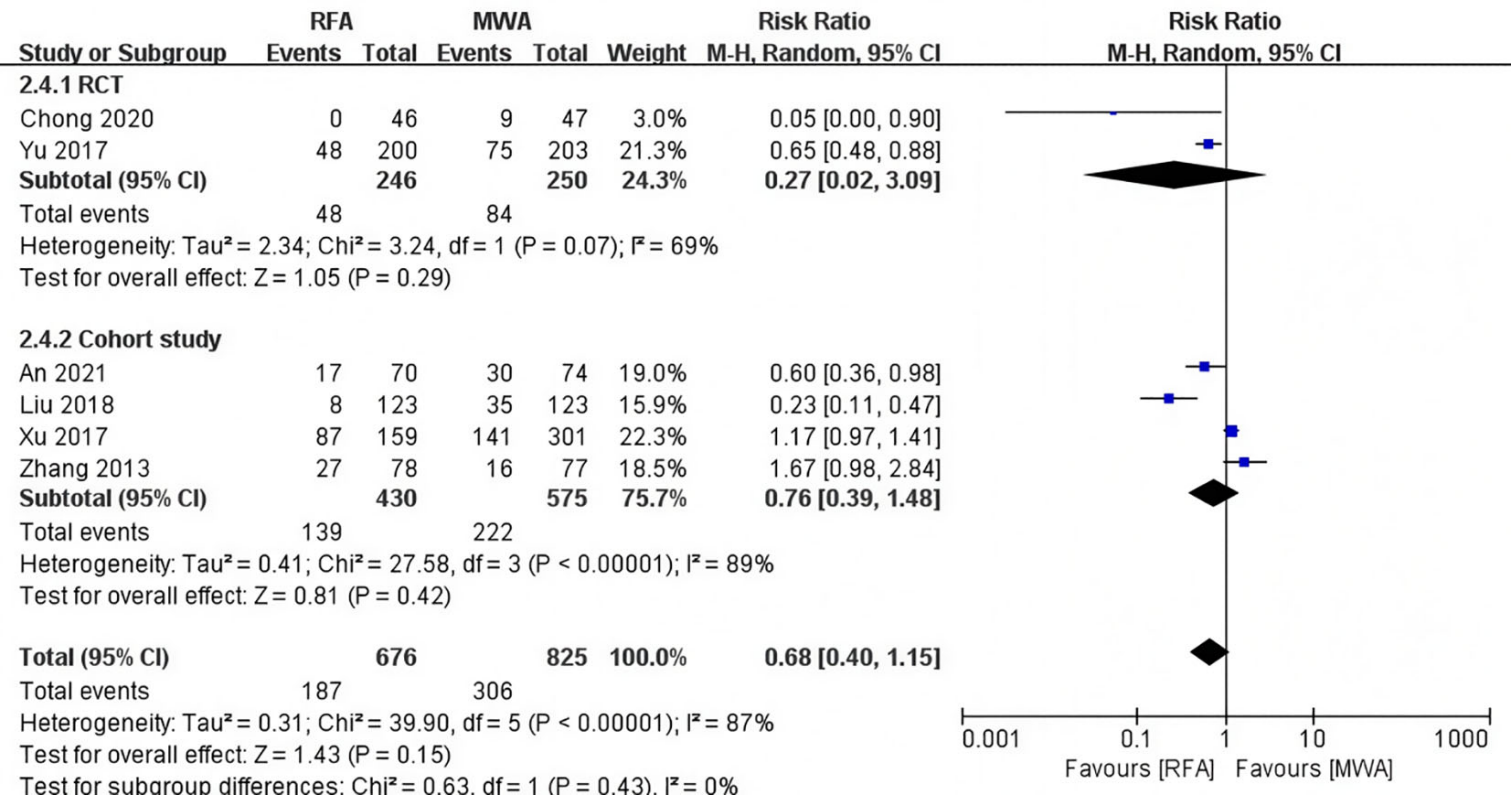
Subgroup analysis of 5-year DFS.

For 5-year DFS (**Figure 9**), the RCT subgroup included two studies. The pooled results showed no statistically significant difference, with a confidence interval crossing 1. Moderate heterogeneity was observed within this subgroup (I²=69%, P = 0.07). The cohort study subgroup incorporated four studies. The pooled analysis also suggested no significant difference, but the heterogeneity within this subgroup was substantial (I²=89%, P<0.01). Consequently, the overall pooled analysis combining both subgroups still indicated no statistically significant difference in 5-year DFS, with similarly high overall heterogeneity (I²=87%, P<0.01). In contrast, the test for subgroup differences again showed no statistically significant variation in effect trends between the study designs (P = 0.43, I²=0%).

### Results of the certainty of evidence

The certainty of evidence for six outcome measures was independently evaluated using the GRADE system. This assessment was based on five key domains: risk of bias, inconsistency, indirectness, imprecision, and publication bias. The final certainty levels were categorized as high, moderate, low, or very low (**Table 3**).

**Table 3:** GRADE evidence certainty assessment for core outcome measures.

Outcome measure	Risk of bias	Inconsistency	Indirectness	Imprecision	Publication bias	Overall certainty
CR	Moderate: RCTs had low-to-moderate risk; cohort studies were of high quality. However, one study exhibited high risk due to high attrition. Removing this study enhanced result stability	Low-Moderate: I²=41% (moderate heterogeneity). After removing the source study in sensitivity analysis, I² decreased to 8%, indicating the heterogeneity impact was mitigated	Low: Population, interventions, and outcomes matched the review objective. No indirectness issues were identified	Low: The 95% CI for the pooled RR (0.98, 1.01) was narrow. Seventeen included studies provided a sufficient sample size for precise estimation	Low: The funnel plot was symmetrical, and Egger’s test yielded P = 0.7404 (P>0.05), indicating no significant publication bias	High
1-year OS	Low-Moderate: RCTs exhibited low-to-moderate risk; all cohort studies were of high quality without major sources of bias	Moderate: Original I²=48% (moderate heterogeneity). After excluding a high-risk study, I²=0%, suggesting heterogeneity was attributable to risk of bias	Low: Population, interventions, and outcomes matched the review objective. No indirectness issues were identified	Low: The 95% CI for the pooled RR (0.95, 0.99) was narrow. Ten included studies provided an adequate sample size for good precision	Moderate: The initial funnel plot was asymmetrical (Egger’s test P = 0.0193). After removing the outlier study, the risk of bias was significantly reduced (P = 0.1471)	Moderate
1-year DFS	Low-Moderate: RCTs had low-to-moderate risk; cohort studies were of high quality without major biasing factors	Moderate: I²=58% (moderate-to-high heterogeneity). Following sensitivity analysis, I²=3%. This heterogeneity was explained by the effect magnitude of a single study	Low: Population, interventions, and outcomes matched the review objective. No indirectness issues were identified	Low: The 95% CI for the pooled RR (0.90, 1.04) was reasonable. Seven included studies met the sample size requirements for analysis	Low: The funnel plot was symmetrical, and Egger’s test yielded P = 0.6825 (P>0.05), indicating no significant publication bias	
5-year OS	Low-Moderate: RCTs had low-to-moderate risk; cohort studies were of high quality without major biasing factors	Moderate: I²=47% (moderate heterogeneity). After exclusion in sensitivity analysis, I²=36%, indicating acceptable residual heterogeneity	Low: Population, interventions, and outcomes matched the review objective. No indirectness issues were identified	Low: The 95% CI for the pooled RR (0.93, 1.05) was narrow. Eight included studies provided a sufficient sample size for stable estimation	Low: The funnel plot was symmetrical, and Egger’s test yielded P = 0.7451 (P>0.05), indicating no significant publication bias	Moderate
5-year DFS	Low-Moderate: RCTs and cohort studies collectively had a low risk of bias, but the total number of studies was limited (n=5)	High: I²=81% (high heterogeneity). Sensitivity analysis failed to substantially reduce heterogeneity. The limited number of studies amplified the influence of individual study effects	Low: Population, interventions, and outcomes matched the review objective. No indirectness issues were identified	High: The 95% CI for the pooled RR (0.52, 1.33) was wide. With only five included studies, the limited sample size resulted in insufficient precision	High: The funnel plot was asymmetrical, and Egger’s test yielded P = 0.0381 (P<0.05), indicating significant small-study effects. The low number of studies also limited the test’s power	Low
MAE	Low-Moderate: RCTs exhibited low-to-moderate risk; all cohort studies were of high quality without major sources of bias	Low: I²=0% (no heterogeneity). Results across 17 included studies were highly consistent	Low: The definition of adverse events was uniform. Interventions and population were well-matched, with no indirectness	Low: Although event numbers were low in some studies, the overall sample size was adequate. The 95% CI for the pooled RR (0.69, 1.27) was reasonably estimated	Low: The funnel plot showed typical symmetry. Egger’s test yielded P = 0.7016 (P>0.05), indicating no significant publication bias	High

## Discussion

### Comprehensive interpretation of results

This systematic review and meta-analysis integrated 19 clinical studies to compare the clinical value of image-guided RFA and MWA for treating small-to-medium HCC, focusing on core outcomes including CR, survival endpoints, and MAEs. The primary conclusion is that no statistically significant differences exist between the two ablation techniques in terms of efficacy and safety, indicating their comparable clinical utility. However, caution is warranted when interpreting the results for 5-year DFS due to high heterogeneity.

Regarding the key efficacy endpoint of CR, the pooled analysis showed no significant difference between RFA and MWA. The results were robust, with moderate heterogeneity that was largely explained by a single study in sensitivity analysis, and the overall certainty of evidence was rated high per GRADE. This equivalence is attributable to their core therapeutic principles: both rely on local hyperthermia to destroy tumor tissue—RFA via the thermal effect of alternating electrical current and MWA via frictional heat generated by molecular agitation (31). In the context of treating small-to-medium HCC, both techniques can precisely control the thermal field under image guidance to ensure complete tumor coverage, thereby yielding comparable immediate efficacy.

An initial analysis suggested a 1-year OS advantage for MWA, but this finding disappeared after excluding a high-risk-of-bias study, indicating that the apparent difference was likely driven by methodological flaws rather than genuine efficacy. For 1-year DFS, no significant difference was observed, and the observed moderate-to-high heterogeneity was largely explained by a single study. Clinically, short-term survival after ablation depends more on baseline liver function reserve, tumor burden, and adherence to post-procedural surveillance than on the choice of ablation device. Both RFA and MWA effectively eradicate the index tumor and provide comparable early tumor control.

For 5-year OS, the two techniques provided comparable benefits with consistent results across studies. For 5-year DFS, no significant difference was found; however, this analysis was limited by high heterogeneity and low statistical power due to the small number of included studies (n=5). The high heterogeneity likely reflects variations in patient populations, tumor characteristics, and follow-up protocols across studies. Consequently, the 5-year DFS finding should be considered exploratory, and no firm clinical recommendation can be made based on this outcome alone.

The incidence of MAEs did not differ significantly between RFA and MWA, with no heterogeneity across studies and stable sensitivity analysis results. Both procedures are minimally invasive and share similar access routes. Complications primarily arise from puncture-related injury or damage to adjacent structures. With image guidance, operators can adjust probe placement to avoid critical structures. This likely explains the comparable safety profiles. Clinically, the choice between RFA and MWA for safety reasons should focus on specific anatomical challenges and operator familiarity with each device, rather than assuming inherent superiority of one technique.

The observed heterogeneity across studies, particularly for DFS outcomes, likely stems from multiple clinical factors. First, tumor size and location may influence outcomes, as perivascular tumors could be more challenging for RFA due to blood flow, whereas subcapsular tumors may pose different risks for thermal injury. Second, technical variations within RFA (conventional, multi-bipolar, no-touch) and MWA (conventional, cooled-shaft antenna) may affect ablation zone geometry, but these details were inconsistently reported. Third, operator expertise and institutional volume affect complication rates and local tumor control, yet most studies did not adjust for these factors. Fourth, follow-up protocols (imaging modality, interval, definition of recurrence) varied considerably, contributing to heterogeneity in DFS. Therefore, rather than declaring one technique superior, clinical decision-making should be individualized. RFA remains an excellent choice with a longer track record and extensive published evidence, especially for typical small HCCs away from large vessels. MWA may offer advantages for larger tumors (within the ≤5 cm range), tumors adjacent to vessels, or when faster ablation is desired. Ultimately, both techniques are valuable tools, and the optimal choice depends on tumor characteristics, available resources, and operator preference.

The novelty lies in four aspects: (1) exclusive focus on the small-to-medium subgroup, which represents the optimal window for local ablative therapy; (2) inclusion of both RCTs and high-quality cohort studies with advanced bias control (e.g., propensity score matching and inverse probability weighting); (3) GRADE certainty assessment for each outcome to guide clinical decision-making; and (4) incorporation of the most recent evidence published up to January 2026, including studies not captured by previous meta-analyses. Based on these updated and more inclusive data, the present study concludes that the core efficacy and safety of RFA and MWA are comparable for small-to-medium HCC, supporting an individualized approach to technique selection based on tumor characteristics, available resources, and operator expertise.

### Limitations

This review has several limitations. First, only six of nineteen included studies were randomized controlled trials; the remainder were cohort studies prone to selection bias. Second, the analysis of 5−year DFS was underpowered due to a small sample size, contributing to high heterogeneity and reduced reliability. Third, technical procedures varied across studies (e.g., different RFA and MWA techniques, imaging guidance modalities), which could not be fully adjusted. Fourth, outcome measurement lacked standardization, as some studies did not specify the imaging modality used for evaluation. Fifth, publication bias was evident for 5−year DFS (Egger’s test P = 0.0381). Sixth, several studies did not report ethnicity, socioeconomic status, or follow−up adherence, limiting generalizability. Seventh, the exclusion of grey literature and lack of regional terminology adjustments may have exacerbated publication bias. Eighth, irregular reporting in primary studies led to missing key parameters, and bias assessment involved subjective judgment. Ninth, time−to−event outcomes were not analyzed using time−series methods, and meta−regression could not be performed due to an insufficient number of studies (e.g., only five for 5−year DFS), precluding quantitative exploration of heterogeneity sources such as tumor size or ablation type. Tenth, secondary outcomes (ablation time, hospital stay) were not analyzed, preventing cost−effectiveness evaluation. Eleventh, the GRADE assessment did not incorporate the minimal clinically important difference for imprecision, and rating all cohort studies as low−quality evidence may have underestimated well−conducted cohort studies.

### Implications for practice, policy, and future research

In clinical practice, the choice between RFA and MWA can be individualized based on patient-specific factors (tumor characteristics, liver function), available institutional equipment, and operator expertise. MWA may offer specific advantages for larger tumors (within the ≤5 cm range) due to a larger ablation zone, for tumors adjacent to vessels due to reduced heat-sink effect, or when faster ablation is desired due to quicker heating. RFA remains a reliable option with a longer track record, especially for typical small HCCs away from large vessels.

From a policy perspective, efforts should be directed toward standardizing the definitions of core efficacy endpoints, follow-up protocols, and criteria for adverse event adjudication. Policies can also promote the rational allocation of ablation therapy resources according to clinical needs, thereby enhancing the homogeneity of regional diagnosis and treatment level.

Future research should prioritize the design and conduct of large-sample, multicenter clinical studies. Standardization of technical ablation details and long-term follow-up protocols is essential. The processes for detecting and adjudicating all outcome measures should be standard to minimize systematic differences between studies. Comprehensive collection of long-term survival and recurrence data is needed to improve the stability of evidence for long-term outcomes. Future studies should also incorporate a broader range of outcome measures, including procedure time, hospital stay, and patient-reported quality of life. Independent subgroup explorations are warranted for patients with tumors in special locations or those with different levels of liver function reserve.

## Conclusion

This systematic review and meta-analysis integrated multiple high-quality clinical studies to systematically compare the clinical application value of image-guided radiofrequency ablation and microwave ablation for treating small-to-medium hepatocellular carcinoma. The results indicate no statistically significant differences between the two ablation techniques in terms of core efficacy indicators and safety, demonstrating their comparable clinical utility. Specifically, tumor complete response rates, short-term and long-term survival benefits, and the risk of major adverse events were all similar. It is important to note that the analysis of long-term disease-free survival was associated with high heterogeneity. Influenced by multiple factors including study design and sample characteristics, conclusions regarding this outcome should be interpreted cautiously. Furthermore, this review has limitations related to the distribution of study designs, standardization of technical procedures, and standard of outcome measurement. In summary, clinical practice can individualize the choice of ablation technique based on tumor characteristics, liver function status, institutional technical capabilities, and operator experience. Both modalities provide reliable local curative treatment for patients with small-to-medium HCC. Future well-designed, large-scale studies are warranted to further validate these conclusions.

## Data Availability

The original contributions presented in the study are included in the article/**Supplementary Material**, further inquiries can be directed to the corresponding author/s.
