# Supplementary material for: Image-guided radiofrequency versus microwave ablation for small to medium hepatocellular carcinoma: a meta-analysis
Source: Front Oncol. 2026 Jul 8;16:1830628. doi: 10.3389/fonc.2026.1830628 (PMC13388185; doi:10.3389/fonc.2026.1830628)
Supplement: Supplementary file 1 [file Table1.docx]

**Electronic Supplementary Material Table S1.** Systematic literature review search terms and strategy.

| **Search terms for PubMed** |
| --- |
| #1 (hepatocellular carcinoma[Mesh] OR HCC[Title/Abstract] OR "small and medium hepatocellular carcinoma"[Title/Abstract]) |
| #2 (radiofrequency ablation[Mesh] OR RFA[Title/Abstract] OR microwave ablation[Mesh] OR MWA[Title/Abstract]) |
| #3 (complete ablation rate[Title/Abstract] OR overall survival rate[Title/Abstract] OR disease-free survival rate[Title/Abstract] OR adverse event[Title/Abstract]) |
| #4 (randomized controlled trial[pt] OR cohort study[pt] OR prospective study[Title/Abstract] OR retrospective study[Title/Abstract]) |
| #1 AND #2 AND #3 AND #4 |
| **Search terms for Embase** |
| #1 hepatocellular carcinoma/ OR HCC/ OR small and medium hepatocellular carcinoma.mp. |
| #2 radiofrequency ablation/ OR RFA/ OR microwave ablation/ OR MWA/. |
| #3 complete ablation rate.mp. OR overall survival rate.mp. OR disease-free survival rate.mp. OR adverse event.mp. |
| #4 randomized controlled trial/ OR cohort study/ OR prospective study/ OR retrospective study/. |
| #1 AND #2 AND #3 AND #4 |
| **Search terms for Web of Science** |
| TS=(hepatocellular carcinoma OR HCC OR small and medium hepatocellular carcinoma) AND TS=(radiofrequency ablation OR RFA OR microwave ablation OR MWA) AND TS=(complete ablation rate OR overall survival rate OR disease-free survival rate OR adverse event) AND TS=(randomized controlled trial OR cohort study OR prospective OR retrospective) |
| **Search terms for Cochrane Library** |
| MeSH Terms: hepatocellular carcinoma, radiofrequency ablation, microwave ablationKey Words: HCC, small and medium hepatocellular carcinoma, RFA, MWA, complete ablation rate, overall survival rate, disease-free survival rate, adverse event, randomized controlled trial, cohort study, prospective study, retrospective studyCombination: hepatocellular carcinoma OR HCC OR small and medium hepatocellular carcinoma AND radiofrequency ablation OR RFA OR microwave ablation OR MWA AND complete ablation rate OR overall survival rate OR disease-free survival rate OR adverse event AND randomized controlled trial OR cohort study OR prospective study OR retrospective study |
